# An Electrochemical Synthesis of Functionalized Arylpyrimidines from 4-Amino-6-Chloropyrimidines and Aryl Halides

**DOI:** 10.3390/molecules16075550

**Published:** 2011-06-29

**Authors:** Stéphane Sengmany, Erwan Le Gall, Eric Léonel

**Affiliations:** Electrochimie et Synthèse Organique, Institut de Chimie et des Matériaux Paris-Est (ICMPE), UMR 7182, CNRS, Université Paris-Est Créteil Val-de-Marne, 2-8 rue Henri Dunant, 94320 Thiais, France

**Keywords:** cross-coupling, electrosynthesis, functionalized pyrimidines, nickel catalysis, sacrificial anode process

## Abstract

A range of novel 4-amino-6-arylpyrimidines has been prepared under mild conditions by an electrochemical reductive cross-coupling between 4-amino-6-chloro-pyrimidines and functionalized aryl halides. The process, which employs a sacrificial iron anode in conjunction with a nickel(II) catalyst, allows the formation of coupling products in moderate to high yields.

## 1. Introduction

The mild and efficient formation of carbon-carbon bonds has always constituted one of the more challenging areas of organic synthesis [1,2,3,4,5]. In this context, the synthesis of diazaaromatic compounds by cross-coupling reactions has been the subject of a tremendous development over the past years. As they represent important structures, widespread in many agrochemicals and biologically active compounds [6,7,8,9,10,11,12,13,14,15,16,17], the preparation of substituted pyrimidines has attracted great attention of the organic community. Therefore, many efforts have been devoted to the arylation of the pyrimidine ring unit by using mostly well established palladium- or nickel-catalyzed processes like the Kumada, Suzuki, Stille, or Negishi couplings [18,19,20,21].

We reported recently the preparation of aryl- and heteroarylpyridazines by an electrochemical cross-coupling procedure employing an iron sacrificial anode [22] and a nickel catalyst [23,24]. In the continuation of this preceding work, we would like to report herein the extension of our electrochemical procedure to the preparation in moderate to high yields of a range of novel 4-amino-6-chloropyrimidines **3**.

## 2. Results and Discussion

Although the preparation of substituted diazaaromatic compounds is of current general interest, we were surprised by the lack of reports on the electrochemical arylation of amino chloropyrimidines. As these compounds represent a relevant class of potent pharmacophores, we decided to turn our attention to the synthesis of 4-amino-6-arylpyrimidines **3**. The implementation of this study was additionally motivated by interesting results, highlighted in our previous reports on the electrochemical arylation of substituted halo-pyridazines, which had revealed the singular reactivity of these substrates. Indeed, it was noticed (and was not explained to date) that the nature of the substituent was of great importance over the coupling efficiency.

**Scheme 1:**
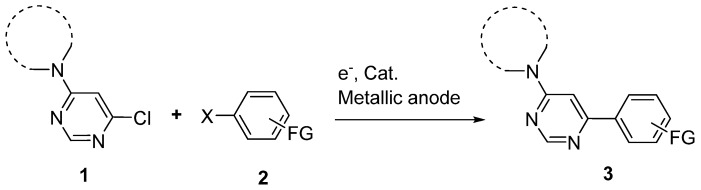
Cross-coupling of 4-amino-6-chloropyrimidine with functionalized aryl halides.

Consequently, we considered that these peculiar features should worth a further fundamental study dedicated to the examination of 4-amino-6-chloropyrimidines behavior in electrochemical couplings. The preparation of 4-amino-6-arylpyrimidines **3** was thus envisaged through a catalyzed reductive cross-coupling reaction between 4-amino-6-chloropyrimidines **1** and aryl halides **2** (Scheme 1).

To this end, we decided to employ an electrochemical method, based on the use of an iron sacrificial anode, which had shown to provide very interesting results on similar reaction systems [25,26]. Few 4-amino-6-chloropyrimidines **1** being commercially available, a range of these starting compounds was initially prepared in high yields by mono-amination of 4,6-dichloropyrimidine using standard procedures [27]. For the implementation of the subsequent electrochemical coupling, preliminary experiments indicated that the most efficient conditions are the following: the reaction is carried out under an inert atmosphere of argon, in undivided cell fitted with an iron rod as the anode, a nickel foam as the cathode, in DMF at room temperature in the presence of nickel bromide 2,2’-bipyridine complex (NiBr_2_bpy) as the catalyst. As reported elsewhere, a pre-electrolysis in the presence of a catalytic amount of 1,2-dibromoethane at 0.2 A during 15 minutes, as well as the use of an overstoichiometric amount of the aromatic halide **2** versus the chloropyrimidine **1**, are required for the efficiency of the coupling. A constant current of 0.2 A [28] is then applied until consumption of the starting chloropyrimidine **1**. 

Using these conditions, 4-amino-6-chloropyrimidines **1** were subjected to electroreductive cross-couplings with diversely functionalized aromatic halides **2**. As they represent an appropriate compromise between commercial availability, cost, and reactivity, bromides were generally used. However, when functional groups borne by the phenyl moieties were electron-donating, fluorine, or hydrogen, the corresponding aryl iodides were used instead of aryl bromides to limit the formation of by-products under these conditions. Results are summarized in Table 1. 

The cross-coupling proceeds in moderate to good yields. The nature (electron-donating, electron-withdrawing, halide atoms) and the position (*ortho*, *meta* and *para*) of the substituents borne by the phenyl do not have a notable influence on the coupling when aliphatic or alicyclic amines are linked to the pyrimidine ring (Table 1, entries 1–4), except when the substituent of the phenyl is at the *ortho* position. Indeed, in this case, the yield seems to be more limited, presumably due the steric hindrance of the substituent (Table 1, entry 3). 

Surprisingly, a similar decrease of the yields was also observed with chloropyrimidines substituted at the 4-position by some aromatic amine such as imidazole, pyrrole or 4-methoxyaniline (Table 1, entries 6, 7 and 8). In the case of imidazole and pyrrole (Table 1, entries 6 and 7), the moderate yields can be attributed to a rapid consumption of a part of the starting chloropyrimidines **1** by dimerization, despite the presence of an excess of the aryl halide in the medium. With 4-methoxyaniline as the amine (Table 1, entry 8), the coupling reaction is very slow compared to side reactions, mainly corresponding to the reduction of the aryl halide, even in the presence of a large excess of this latter. However, the corresponding coupling product is of particular interest as the further deprotection of the PMP group [29] should give rise to the formation of a –NH_2_ substituent at the 4-position. In addition, this latter result is very encouraging since it constitutes, to the best of our knowledge, the first example of an electrochemical cross-coupling involving a diazaaromatic substrate bearing an unprotected N-H substituent. This is of particular significance for further potential transformations of the resulting compounds by N-C bond formations. Additionally, it also constitutes an entry to even more interesting stereoselective additions and functional group interconversions by using, for instance, a-amino esters connected to the pyrimidine moiety. This assertion was partly verified by an experiment involving a chloropyrimidine substituted with a secondary amino ester, L-proline methyl ester (Table 1, entry 9). Although an only limited yield was obtained, this constituted the experimental evidence that the aliphatic CO_2_Me group is compatible with this electrochemical process.

In all the cases, an excess of the aryl halide **2** was used to counterbalance the consumption of a part of the halide by dimerization. Furthermore, it should be noted that for couplings where a rapid consumption of aryl halide is observed (Table 1, entries 2, 3, 4, 8 and 9), either by reduction of C-halogen bond into C-H, or by dimerization, one additional equivalent of aryl halide is introduced during the reaction to drive the coupling to completion.

**Table 1:** Cross-coupling of 4-amino-6-chloropyrimidines with various functionalized aryl halides. 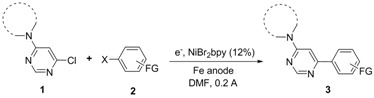

Entry	Substrate	Halide	Reaction Time	Product	Isolated Yield (%)
1	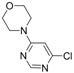	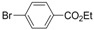	5 h	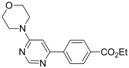 **3a**	83
2	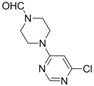	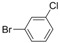	5 h	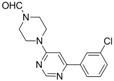 **3b**	78^a^
3	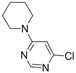	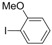	6 h	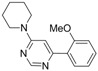 **3c**	48^b^
4	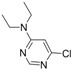	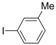	5 h 30	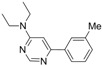 **3d**	99^b^
5	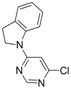	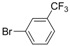	6 h 30	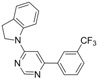 **3e**	77
6	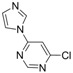	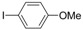	4 h 30	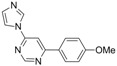 **3f**	36
7	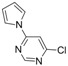	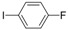	3 h 30	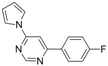 **3g**	34
8	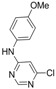	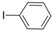	6 h 15	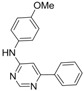 **3h**	41^b^
9	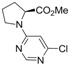	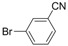	6h	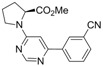 **3i**	41^b^

^a^ After 4h of reaction, one more equivalent of ArX was added; ^b^ After 2 h 30, one more equivalent of ArX was added.

**Scheme 2:**
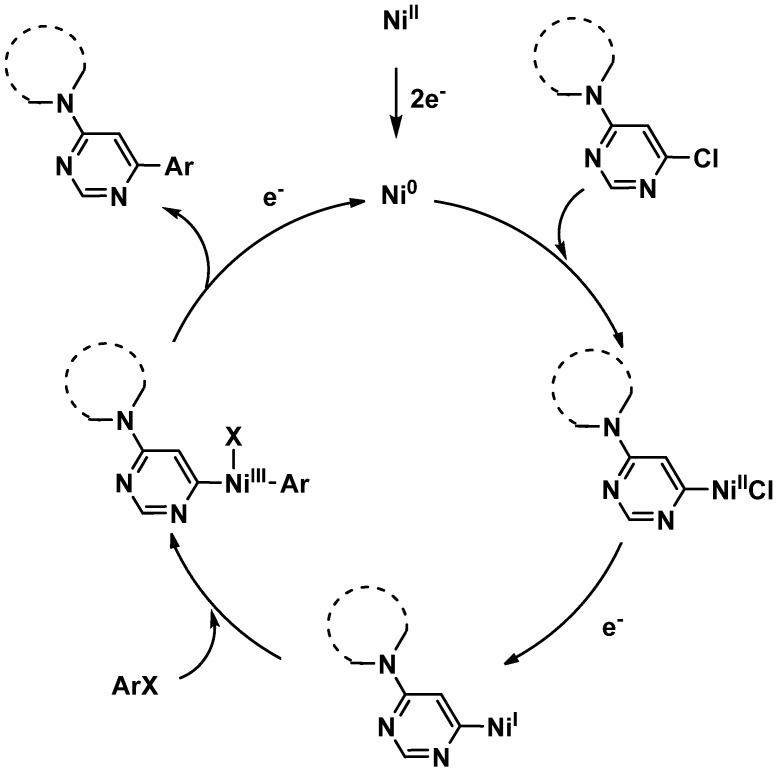
Proposed reaction mechanism.

The mechanism of this cross-coupling reaction might be similar to those already described for this type of electrochemical syntheses [23] (Scheme 2). Accordingly, the electrocatalytic cycle would be initiated by a preliminary reduction of Ni(II) into Ni(0). At this stage, an oxidative addition of Ni(0) into the C-X bond of either chloropyrimidine or aryl halide should be envisaged. A recent electroanalytical work dedicated to the mechanistical investigation of cross-coupling reactions involving chloropyridazine [24] has reported that the oxidative addition proceeds faster with the chloropyridazine than with the aryl halide. This should also be likely the case with pyrimidines derivatives, which should give rise to the formation of HetArNi^II^Cl species. The monoelectronic reduction of this complex should provide a HetArNi^I^ species which might be involved in a further oxidative addition onto the aryl halide, furnishing a HetArNi^III^(Ar)X complex. A reductive elimination on this latter should give rise to the formation of the final coupling product along with Ni^I^X, allowing, after electrochemical reduction, the recovery of Ni^0^ which can be reengaged in the catalytic cycle.

The efficiency of the procedure proved to be sensitive to the applied current intensity. Consequently, a 0.2 A constant current intensity was found to be the best compromise in terms of reaction efficiency and rapidity. Indeed, it is well recognized [28] that a lower intensity encourages a metathesis of HetArNi^II^Cl providing HetAr_2_Ni^II^ and NiCl_2_, which results in the partial loss of both the catalyst and the heteroaromatic chloride by reductive elimination. On the contrary, a higher intensity promotes the reduction of HetArNi^II^Cl into HetArNi^I^ and Cl^−^, thus encouraging the formation of HetArNi^III^(Ar)X by a further Ni(I) insertion into the C-X bond of ArX, which is the desired pathway of the reaction.

## 3. Experimental

### 3.1. General

Solvents and reagents were purchased from commercial suppliers and used without further purification. Nickel bromide 2,2’-bipyridine complex (NiBr_2_bpy) was prepared from nickel bromide hydrate and 2,2’-bipyridyl [30]. Reactions were monitored by gas chromatography (GC) using a Varian 3900 chromatograph fitted with an SGE capillary column (*l *= 5m, *Ø = *0.32mm, *df* = 0.5µm). Melting points were measured on a Büchi B-545 apparatus. Infrared spectra were recorded in ATR mode on a Bruker TENSOR 27 spectrometer and treated via OPUS software. NMR spectra were recorded in CDCl_3_ at 400 MHz (^1^H), 100 MHz (^13^C) and 376 MHz (^19^F) on a Bruker Avance II 400 spectrometer. Chemical shift (δ) are reported in parts per million (ppm) relative to the residual solvent signal. Coupling constant values (J) are given in Hertz (Hz) and refer to apparent multiplicities, indicated as follows: s (singlet), d (doublet), t (triplet, q (quartet), m (multiplet). Mass spectra (in electronic impact (EI^+^) ionization mode) were measured on a Thermo Scientific ITQ 700 GC-MS spectrometer fitted with a Varian column (*l* = 25m, *Ø = *0.25mm, *df* = 0.25µm). High Resolution Mass Spectra (HRMS) were performed in positive Electrospray Ionisation (ESI^+^) mode by the Mass Spectrometry service of the ICOA (Institut de Chimie Organique et Analytique), Orléans, France. Purifications were performed by flash chromatography on silica gel (granulometry 70–200 µm). Compounds which have been previously described in the literature are linked to the corresponding bibliographic references whereas compounds labelled by asterisk (*) are, to the best of our knowledge, new compounds. The asterisk does not correspond to a footnote

### 3.2. Typical Procedure for the Cross-Coupling Reactions

To an undivided electrochemical cell, fitted by an iron rod as the anode and surrounded by a nickel foam as the cathode, were added DMF (50 mL), tetra*-n*-butylammonium bromide (0.5 mmol, 400 mg), and 1,2-dibromoethane (2.6 mmol, 215 µL). The mixture was electrolyzed under argon at a constant current intensity of 0.2 A for 15 min at room temperature. Then the current was stopped, and NiBr_2_bpy (0.5 mmol, 187 mg), the 4-amino-6-chloropyrimidine **1** (4 mmol), and the substituted aromatic halide (8 mmol) were sequentially added. The solution was electrolyzed at 0.2 A until the starting 4-amino-6-chloropyrimidine was totally consumed (3h30–6h). A saturated EDTA solution (50 mL) was added, and the resulting solution extracted with CH_2_Cl_2_ containing 2–5% of methanol (4 × 50 mL). The combined organic layers were dried over MgSO_4_, filtered, and evaporated under vacuum. The crude product was purified by flash chromatography on silica (granulometry 70-200 µm), eluted with a gradient mixture of solvents (pentane/Et_2_O, or pentane/acetone for some polar cross-coupling compounds).

Ethyl 4-(6-morpholinopyrimidin-4-yl)benzoate* (**3a**). White crystals. mp: 130–131 °C (pentane/diethyl ether). ATR-FTIR (neat, cm^−1^): 2955, 2862, 1702, 1587, 1570, 1277, 1245, 1109, 983. ^1^H-NMR (400 MHz): δ 8.72 (s, 1H), 8.13 (d, *J* = 8.3 Hz, 2H), 8.03 (d, *J* = 8.3 Hz, 2H), 6.91 (s, 1H), 4.41 (q, *J *= 7.2 Hz, 2H), 3.82 (t, *J* = 4.9 Hz, 4H), 3.72 (t, *J* = 4.9 Hz, 4H), 1.42 (t, *J* = 7.2 Hz, 3H). ^13^C-NMR (100 MHz): δ 164.3, 160.8, 160.5, 156.7, 140.2, 129.9, 128.1, 125.0, 97.2, 64.6, 59.3, 42.3, 12.5_._ MS, m/z (%): 314 (16), 313 ([M]**^.^**^+^, 81), 312 (40), 285 (7), 284 (15), 283, (46), 282 (100), 270 (31), 269 (13), 268 (38), 257 (13), 256 (63), 255 (33), 254 (19), 240, (13), 228 (23), 210 (9), 155 (11), 146 (8), 128 (9). HRMS Calcd. for C_17_H_20_N_3_O_3_ [M+H]^+^: 314.14492, found [M+H]^+^: 314.14997.

4-[6-(3-Chlorophenyl)pyrimidin-4-yl]piperazine-1-carbaldehyde* (**3b**). Pale yellow amorphous solid.mp: 123–125 °C (pentane/acetone). ATR-FTIR (neat, cm^−1^): 2859, 1664, 1587, 1567, 1434, 1228, 981, 695. ^1^H-NMR (400 MHz): δ 8.72 (s, 1H), 8.16 (s, 1H), 7.97 (s, 1H), 7.86 (d, *J *= 7.1 Hz, 1H), 7.44–7.39 (m, 2H), 6.89 (s, 1H), 3.86-3.83 (m, 2H), 3.75–3.71 (m, 2H), 3.71–3.67 (m, 2H), 3.53–3.49 (m, 2H). ^13^C-NMR (100 MHz): δ 162.5, 162.4, 160.9, 158.5, 139.7, 134.9, 130.2, 130.1, 127.1, 125.1, 98.8, 45.0, 44.5, 43.6, 39.7_._ MS, m/z (%): 304 (11), 303 (8), 302 ([M]**^.^**^+^, 33), 301 (9), 246 (9), 245 (7), 244, (24), 234 (27), 233 (25), 232 (46), 231 (72), 230 (42), 221 (5), 220 (32), 219 (15), 218 (100), 208 (7), 207 (6), 206 (15), 190 (6), 189 (6), 162 (13), 155 (6), 138 (6), 136 (13), 127 (12), 68 (5). HRMS Calcd. for C_15_H_16_ClN_4_O [M+H]^+^: 303.10072, found [M+H]^+^: 303.10078.

4-[2-Methoxyphenyl)-6-(piperidin-1yl)pyrimidine* (**3c**). Brown oil. ATR-FTIR (neat, cm^−1^): 2934, 2852, 1585, 1572, 1492, 1240, 1023, 979, 752. ^1^H-NMR (400 MHz): δ 8.60 (s, 1H), 7.74 (d, *J *= 7.2 Hz, 1H), 7.29 (t, *J *= 7.5 Hz, 1H), 7.02-6.96 (m, 2H), 6.91 (d, *J *= 8.1 Hz, 1H), 3.79 (s, 3H), 3.59–3.55 (m, 4H), 1.64-1.54 (m, 6H). ^13^C-NMR (100 MHz): δ 161.8, 161.3, 158.1, 157.3, 130.8, 130.5, 127.9, 121.0, 111.5, 103.3, 55.8, 45.1, 25.5, 24.7_._ MS, m/z (%): 270 (14), 269 ([M]**^.^**^+^, 92), 268 (100), 241 (20), 240 (52), 226 (11), 225 (21), 224 (27), 223 (12), 214 (12), 213 (14), 212 (6), 211 (11), 210 (14), 199 (8), 198 (10), 197 (20), 196 (7), 186 (7), 185 (12), 184 (6), 183 (6), 171 (6), 164 (14), 157 (6), 156 (8), 131 (18), 84 (8). HRMS Calcd. for C_16_H_20_N_3_O [M+H]^+^: 270.16009, found [M+H]^+^: 270.16026

N,N-Diethyl-6-(meta-tolyl)pyrimidin-4-amine* (**3d**). Colourless oil. ATR-FTIR (neat, cm^−1^): 2972, 2928, 1577, 1509, 1352, 1255, 1026, 980, 791. ^1^H-NMR (400 MHz): δ 8.69 (s, 1H), 7.81 (s, 1H), 7.73 (d, *J* = 7.5 Hz, 1H), 7.36 (t, *J *= 7.5 Hz, 1H), 7.26 (d, *J *= 7.5 Hz, 1H), 6.74 (s, 1H), 3.60 (q, *J *= 7.0 Hz, 4H), 2.44 (s, 3H), 1.25 (t, *J *= 7.1 Hz, 6H). ^13^C-NMR (100 MHz): δ 162.8, 161.3, 158.5, 138.5, 138.4, 130.6, 128.5, 127.6, 123.9, 98.1, 42.3, 21.5, 12.8_._ MS, m/z (%): 242 (15), 241 ([M]**^.^**^+^, 74), 240 (14), 226 (26), 213 (17), 212 (100), 199 (9), 198 (62), 197 (8), 142 (7), 116 (7), 115 (18). HRMS Calcd. for C_15_H_20_N_3_ [M+H]^+^: 242.16517, found [M+H]^+^: 242.16525.

1-[6-(3-(Trifluoromethyl)phenyl)pyrimidin-4-yl]indoline* (**3e**). Pale yellow amorphous solid. mp:154–155 °C (pentane/diethyl ether). ATR-FTIR (neat, cm^−1^): 3044, 2931, 1573, 1496, 1261, 1113, 1092, 1071, 754, 668. ^1^H-NMR (400 MHz): δ 8.90 (s, 1H), 8.48 (d, *J* = 7.5 Hz, 1H), 8.31 (s, 1H), 8.22 (d, *J *= 7.1 Hz, 1H), 7.75 (d, *J *= 7.2 Hz, 1H), 7.63 (t, *J* = 7.5 Hz, 1H), 7.29–7.23 (m, 2H), 7.01 (t, *J* = 7.1 Hz, 1H), 6.96 (s, 1H), 4.12 (t, *J* = 8.3 Hz, 2H), 3.29 (t, *J *= 8.3 Hz, 2H). ^13^C-NMR (100 MHz): δ 161.8, 159.8, 158.3, 143.3, 138.7, 131.9, 131.4 (q, *J* = 30.0 Hz), 130.2, 129.3, 127.5, 126.8, 124.9, 123.9, 122.7, 116.3, 100.7, 48.7, 27.5_._^19^F-NMR (376 MHz): δ −62.7. MS, m/z (%): 342 (19), 341 ([M]**^.^**^+^, 100), 340 (89), 325 (14), 213 (7), 170 (8), 118 (27), 117 (6), 91 (5). HRMS Calcd. for C_19_H_15_F_3_N_3_ [M+H]^+^: 342.12126, found [M+H]^+^: 342.12134.

4-(1H-Imidazol-1yl)-6-(4-methoxyphenyl)pyrimidine* (**3f**). Pale yellow amorphous solid. mp:183.5–184.5 °C (pentane/acetone). ATR-FTIR (neat, cm^−1^): 2931, 1585, 1485, 1247, 1173, 1023, 832, 580. ^1^H-NMR (400 MHz): δ 9.05 (s, 1H), 8.53 (s, 1H), 8.11 (d, *J* = 8.8 Hz, 2H), 7.76 (s, 1H), 7.58 (s, 1H), 7.26 (s, 1H), 7.05 (d, *J* = 8.8 Hz, 2H), 3.90 (s, 3H). ^13^C-NMR (100 MHz): δ 166.3, 162.6, 159.1, 155.6, 135.2, 131.6, 128.9 128.4, 115.7, 114.5, 102.8, 55.5_._ MS, m/z (%): 253 (17), 252 ([M]**^.^**^+^, 100), 251 (6), 226 (21), 225 (30), 210 (13), 182 (6), 158 (5). HRMS Calcd. for C_14_H_13_N_4_O [M+H]^+^: 253.10839, found [M+H]^+^: 253.10837.

4-(4-Fluorophenyl)-6-(1H-pyrrol-1-yl)pyrimidine* (**3g**). White crystals. mp: 83–84 °C (pentane/diethyl ether). ATR-FTIR (neat, cm^−1^): 3129, 3058, 1587, 1585, 1552, 1483, 1221, 1061, 832, 753, 571. ^1^H-NMR (400 MHz): δ 9.01 (s, 1H), 8.13-8.10 (m, 2H), 7.65–7.63 (m, 2H), 7.53 (s, 1H), 7.26–7.20 (m, 2H), 7.46-7.42 (m, 2H). ^13^C-NMR (100 MHz): δ 164.8 (d, *J* = 252.2 Hz), 164.7, 159.0, 157.5, 132.8 (d, *J* = 2.8 Hz), 129.3 (d, *J* = 8.7 Hz), 118.1, 116.1 (d, *J* = 21.8 Hz), 113.0, 102.6_._^19^F-NMR (376 MHz): δ -109.0. MS, m/z (%): 240 (16), 239 ([M]**^.^**^+^, 100), 238 (17), 213 (20), 212 (37), 211 (11), 146 (9), 126 (10). HRMS Calcd. for C_14_H_11_FN_3_ [M+H]^+^: 240.09315, found [M+H]^+^: 240.09316.

*N-(4-Methoxyphenyl)-6-phenylpyrimidin-4-amine *(**3h**) [31]. Pale yellow crystals. mp: 150–151 °C (pentane/diethyl ether). ATR-FTIR (neat, cm^−1^): 3236, 2995, 1587, 1569, 1505, 1411, 1241, 1033, 990, 828, 767, 688, 609. ^1^H-NMR (400 MHz): δ 8.71 (s, 1H), 7.94–7.91 (m, 2H), 7.85 (s, 1H), 7.7–45–7.43 (m, 3H), 7.32–7.28 (m, 2H), 6.99–6.96 (m, 3H), 3.86 (s, 3H). ^13^C-NMR (100 MHz): δ 163.7, 162.6, 158.8, 157.6, 137.6, 130.8, 130.3, 128.7, 126.9, 125.6, 114.9, 99.0, 55.6_._ MS, m/z (%): 278 (19), 277 ([M]**^.^**^+^, 100), 276 (67), 263 (9), 262 (50), 261 (7), 235 (5), 234 (10), 207 (5), 128 (11), 108 (10). HRMS Calcd. for C_17_H_16_N_3_O [M+H]^+^: 278.12879, found [M+H]^+^: 278.12892.

(S)-Methyl 1-[6-(3-cyanophenyl)pyrimidin-4-yl]pyrrolidine-2-carboxylate* (**3i**). Pale yellow amorphous solid. mp: 102-106 °C (pentane/diethyl ether). ATR-FTIR (neat, cm^−1^): 2952, 2875, 2359, 2229, 1739, 1587, 1502, 1207, 1168, 798, 693, 675. ^1^H-NMR (400 MHz): δ 8.57 (s, 1H), 8.20 (s, 1H), 8.14 (d, *J* = 7.0 Hz, 1H), 7.65 (d, *J* = 7.6 Hz, 1H), 7.51 (t, *J* = 7.6 Hz, 1H), 6.64 (s, 1H), 4.67 (broad s, 1H), 3.69 (s, 4H), 3.48 (broad s, 1H), 2.28-2.25 (broad m, 1H), 2.12-2.08 (broad m, 3H). ^13^C-NMR (100 MHz): δ 173.3, 160.4, 158.5, 139.3, 133.2, 131.2, 130.7, 129.6, 118.5, 113.0, 99.5, 59.4, 52.4, 46.8, 29.8, 24.0_._ MS, m/z (%): 308 ([M]**^.^**^+^, 14), 250 (17), 249 (100), 153 (8). HRMS Calcd. for C_17_H_17_N_4_O_2_ [M+H]^+^: 309.13460, found [M+H]^+^: 309.13467.

## 4. Conclusions

In summary, we report in this paper the nickel-catalyzed electrochemical cross-couplings of a range of 4-amino-6-chloropyrimidines with diversely substituted aryl halides. The method provides an efficient entry to some novel 4-amino-6-arylpyrimidines, in satisfactory to high yields. The great functional tolerance of the reaction, as well as the possibility of engaging chloropyrimidines bearing N-H derivatives in the coupling, opens the way to further subsequent transformations by N-C couplings and functional group interconversions. The extension of the reaction to heteroaromatic halides in currently in progress and will be reported in due course.
